# Calcium Ion Channels: Roles in Infection and Sepsis Mechanisms of Calcium Channel Blocker Benefits in Immunocompromised Patients at Risk for Infection

**DOI:** 10.3390/ijms19092465

**Published:** 2018-08-21

**Authors:** John A. D’Elia, Larry A. Weinrauch

**Affiliations:** E P Joslin Research Laboratory, Kidney and Hypertension Section, Joslin Diabetes Center, Department of Medicine, Mount Auburn Hospital, Harvard Medical School, Boston and Cambridge, 521 Mount Auburn Street Watertown, MA 02472, USA; jd’elia@joslin.harvard.edu

**Keywords:** calcium ion channels, calcium channel blockade, sepsis, infection, immunosuppression

## Abstract

Immunosuppression may occur for a number of reasons related to an individual’s frailty, debility, disease or from therapeutic iatrogenic intervention or misadventure. A large percentage of morbidity and mortality in immunodeficient populations is related to an inadequate response to infectious agents with slow response to antibiotics, enhancements of antibiotic resistance in populations, and markedly increased prevalence of acute inflammatory response, septic and infection related death. Given known relationships between intracellular calcium ion concentrations and cytotoxicity and cellular death, we looked at currently available data linking blockade of calcium ion channels and potential decrease in expression of sepsis among immunosuppressed patients. Notable are relationships between calcium, calcium channel, vitamin D mechanisms associated with sepsis and demonstration of antibiotic-resistant pathogens that may utilize channels sensitive to calcium channel blocker. We note that sepsis shock syndrome represents loss of regulation of inflammatory response to infection and that vitamin D, parathyroid hormone, fibroblast growth factor, and klotho interact with sepsis defense mechanisms in which movement of calcium and phosphorus are part of the process. Given these observations we consider that further investigation of the effect of relatively inexpensive calcium channel blockade agents of infections in immunosuppressed populations might be worthwhile.

## 1. Introduction

Immunosuppression may occur for a number of reasons related to an individual’s frailty, debility, disease or from therapeutic iatrogenic intervention or misadventure. A large percentage of morbidity and mortality in immunodeficient populations is related to an inadequate response to infectious agents with slow response to antibiotics, enhancements of antibiotic resistance and markedly increased prevalence of acute inflammatory response, septic and infection related death. Given the known relationships between intracellular calcium ion concentrations, cytotoxicity and cellular death, we review currently available data linking blockade of calcium ion channels and potential decrease in expression of sepsis among immunosuppressed patients. We consider possibilities for therapeutic interference with calcium ion channels that may alter immune responses to invading organisms in immunocompromised populations (Table 1).

## 2. Calcium, Calcium Channel, Vitamin D Mechanisms Associated with Sepsis

In a survey of the United Kingdom General Practice database (Table 2) over 500,000 patients were listed as hypertensive. In this survey, use of angiotensin converting enzyme inhibitors was associated with a significantly higher rate of hospitalization for sepsis as well as mortality at thirty days when compared to use of angiotensin receptor blockers or calcium channel blockers [1]. One retrospective cohort study revealed that when 387 patients hospitalized for pneumonia who had not been treated with calcium channel blockers, were compared to 387 similarly hospitalized patients with pneumonia who had received calcium channel blockers there was a significantly higher incidence of bacteremia, respiratory insufficiency, and transfer to intensive care unit compared among those not so treated [2]. One retrospective evaluation of immunosuppressed recipients of kidney allografts from the pre-angiotensin receptor blocker era noted significantly higher incidence of sepsis and shorter survivals of allograft function for 33 patients who had not received calcium channel blockers as opposed to 36 who did receive calcium channel blockers [3].

Since the patients reviewed in studies of angiotensin-active medications and calcium channel blockers would have had hypertension, a precise mechanism for protection from sepsis would have to include protection from injury to blood vessels supplying skin, bronchus, urinary bladder unless there had been a blood vessel indwelling catheter-line, suggesting the vascular protection hypothesis might be too narrow. Consequently, researchers have developed other approaches. An experimental model for testing the impact of calcium channel blockers on sepsis involving ligation of the cecum with puncture of the wall of the intestine was one such example. This septic shock model was used to [4] demonstrate a lesser accumulation of oxygen radicals [5] as well as longer survival if diltiazem were injected prior to the onset of septic shock.

### 2.1. Positive Results

Several studies have addressed the use of dihydropyridine and non-dihydropyridine calcium channel blockers in the peri-operative period and in longer-term follow up (Table 2), demonstrating improved allograft function [6,7] that did not consistently appear to be secondary to blood pressure control [8,9]. One study did demonstrate stable serum creatinine associated with a fall in renal vascular resistance calculated from mean arterial pressure + renal blood flow [10]. A unique study found the occurrence of acute graft dysfunction (acute tubular necrosis) by biopsy to be significantly lower with verapamil vs. a non-calcium channel medication [11].

Rapid calcium influx into cytoplasm is associated with cell death [12,13] Clinical use for medications affecting calcium channels may involve control of aberrant cardiac rhythm while moderating vascular spasm [14]. As multiple types of calcium ion channels (N-type, L type and T type voltage-dependent calcium channels) became recognizable and multiple pharmaceutical agents with differing action profiles became available, further studies to find a role for these agents were performed.

The fact that some of these agents could directly influence biosynthesis of aldosterone in human adrenocortical cells [15] led to an early interest in their use in hypertension. Animal studies indicated an inhibition of the mineralocorticoid receptor as an additional property of dihydropyridine calcium channel blockers with felodipine being twice as powerful as amlodipine [16]. The other classes of calcium channel blockers (diltiazem, verapamil) were not effective here. Medium-sized blood vessels experience pulsatile flow as pressure is conveyed down to points of resistance and reflected backwards. Arterioles experience non-pulsatile constant flow, which can be increased with vasoconstriction or decreased with vasodilatation. Nitric oxide regulates arteriole flow while calcium-activated potassium channels regulate conduit artery flow [17]. Pathological concentrations of the vasoconstrictor angiotensin can cause damage to the muscular layer of larger blood vessels and to the endothelium of smaller blood vessels [18].

Uncontrolled hypertension may result in loss of vascular integrity with impaired endothelial resistance to sepsis [19,20] through activation of inflammation cascades which inhibit expression of nitric oxide synthase as well as the loss of control of glucose disposal such that oxidative stress disrupts calcium/potassium vascular physiology. These same mechanisms are operative in chronic loss of kidney function through injury to blood vessels and interstitial matrix, which diminishes production of 1,25(OH)_2_ Vitamin D (calcitriol) in renal proximal tubules. Since calcitriol has been shown to be additive to the inotropic effects of norepinephrine and vasopressin [21] and since receptors for 1,25(OH)_2_ D are found in increased amounts in hypertrophied heart muscle [22], the fact that calcitriol synthesis in the proximal tubule decreases as kidney function is lost may be seen as protective from accelerated hypertension. Since vitamin D is useful in controlling excessive secretion of parathyroid hormone in kidney failure, replacement therapy is routine in hemodialysis units. But a further benefit may be protection from sepsis as found in experimental model studies [23,24,25,26].

Initial enthusiasm for calcium channel blockers in renal transplantation related to their role in control of hypertension as well as the possibility that calcium channel blockers might be organ protective from intracellular calcium infusion related cell death (as seen in the necrotic myocardium during acute coronary occlusion). Then chronic myocardial stress in a rat hypertension model demonstrated protection by a calcium channel blocker (mibefradil) from myocardial scarring with an increase in interstitial collagen thought to be the result of increased fibroblast collagen production [27]. But since outcome studies had not shown long-term benefits of vasodilation following myocardial infarction, there was concern for perioperative hypotension with acute kidney allograft injury, which eventually became an impediment to the study of calcium channel blockers in renal transplant centers, particularly as the potential treatment population has aged from less than 55 to greater than 75 years of age under certain circumstances.

Thus, observations connecting calcium channel blockers with prolonged survival of kidney transplant allografts may have been due to both preservation of circulation [8,9,10] to the allograft at risk for ischemia due to the vasoconstrictive effect of the immunosuppressive agent, cyclosporine, but perhaps also to inhibition of mechanisms in the immunological rejection process [6,7]. An unanswered question in multiple centers from several countries is the method of preservation of donor kidneys at a point in time after nephrectomy when they are most susceptible to acute injury. A powerful vasodilator, the calcium channel blocker, lidoflazine, was shown to protect Lewis rat donor kidneys preserved in University of Wisconsin perfusate [28]. Additive effects of cyclosporine and verapamil on suppression of cytotoxic T lymphocyte cell proliferation [29] are considered to be operating via the calcium/calmodulin/calcineurin pathway to nuclear direction of the expression of antibodies through nuclear factor kappa Beta. Additive effects of cyclosporine and verapamil may also be found in suppression of the generation of cytokines by lymphocytes through inhibition of the incorporation of thymidine into DNA or uridine into RNA or leucine into protein.

Eventually the possibility that calcium channel blockers have targets other than on slow calcium channels at the cell borders has been extended to include effects on calcium movement into and out of the cytosol from storage areas within the cell (endoplasmic reticulum, sarcoplasmic reticulum, mitochondria) or on the calcium extrusion technique of pathogen survival in a host whose levels of calcium could be fatal to the pathogen [30]. Our experience with infection in diabetic patients with nephropathy has been limited to end-stage renal disease. Use of peritoneal dialysis for urgent removal of fluid to prevent pulmonary edema [31] or for chronic removal of uremic toxins had been associated with frequent instances of fever, abdominal pain and cloudy peritoneal effluent. When this triad occurred together, the diagnosis of peritonitis with a positive culture of effluent fluid would be expected. However, if only one of the diagnostic pointers were present, there might be some time before a second or third would appear. Our prospective study to determine whether frequent culture of peritoneal dialysis effluent fluid might yield an early warning prior to the clinical syndrome did not produce positive culture results worthy of the extensive expense [32]. At the height of an incursion of illicit distribution of cocaine into the New England sector of the USA, we found the availability of clean needles did not protect diabetic dialysis patients from life-threatening cellulitis, abscess formation, and sepsis [33] All the instances of visceral infection occurred in the setting of cocaine intake. Bacterial infection was detected four times as often in this target population while evidence of spread of hepatitis virus was ten-fold greater amongst users of cocaine. In our studies of cardiovascular end-points in the setting of diabetes associated with cardio-renal complications, there were serendipitous findings in the field of kidney transplantation ten years after the introduction of cyclosporine, which had marked a time of significant improvement in prevention of allograft rejection.

But when our retrospective review of 70 consecutive diabetic recipients of kidney allografts looking for cardiovascular end-points did confirm outcome benefits, it was purely by serendipity that the benefits were mainly derived through protection from attacks of sepsis [3]. Of the 70 study subjects we reviewed from this center, data on 69 was available for analysis. There were 36 individuals who had received calcium channel blockers and 33 who had not received calcium channel blockers. Of the 36, 21 were treated with diltiazem, 10 with nifedipine, and 5 with verapamil. One study subject who initially received nifedipine was subsequently switched to diltiazem. There were no significant outcome differences in cardiovascular events which were found to be less common than sepsis-related events. So, sepsis became the focus of outcome analysis in our retrospective review. Of the 19 individuals with a prior history of cardiovascular events, six who received calcium channel blockers had two sepsis-related events while 13 who had not received calcium channel blockers had 22 sepsis-related events. Of the 50 individuals without a prior history of cardiovascular events, 30 who had received calcium channel blockers had 13 sepsis-related events while 20 individuals who had not received calcium channel blockers experienced 39 sepsis-related events. While these unadjusted differences are highly significant, a weakness of the retrospective analysis would be unequal follow-up times. On the other hand, Kaplan-Meier survival curves were able to demonstrate 1.6 (*p* < 0.02) and 1.5 (*p* < 0.05)-fold greater survivals for patients and allografts, respectively for calcium channel blocker-treated vs non-treated subgroups at 24 months. When both calcium channel blocker and beta blocker medications were used, diabetic non-living allograft kidney recipients having been rigorously evaluated by experienced cardiologists [34] were documented to have durations of survival equivalent to those of non-diabetic recipients of living donor allografts using data from the UNOS Registry [35] and the US Renal Data System [36].

### 2.2. Negative Results

More recently we had the opportunity to perform a post-hoc evaluation of kidney transplantation outcomes in the International Folic Acid for Vascular Outcomes Reduction in Transplant (FAVORIT) trial, finding the incidence of infection risks to be much higher in diabetic recipients of allografts from living or non-living donors [37]. Since follow-up in this study began on average three years post-transplantation surgery, years four to ten were the observation period when most of the acute rejection and cardiovascular issues would probably have been resolved. Thus, the FAVORIT study was useful in documenting the importance of infection as the most serious outcome measure under the unique conditions involved. Of the 4110 individuals recruited into the FAVORIT trial, there were 2447 non-diabetic study subjects of whom 199 (8.2%) died during the study; 166 Type 1 diabetic study subjects of whom 44 (26.5%) died during the study; and 1497 Type 2 diabetic study subjects of whom 250 (16.7%) died during the study. Thus, there were 493 deaths with 191(38.7%) from cardiovascular causes and 286 (58.0%) from non-cardiovascular sources. Of the 286 non-cardiovascular deaths, 113 (59.2%) were due to infection while 76 (39.8%) were due to malignancy associated with immune-suppression.

Our results confirmed those from the UK National Health Service which had reported a 70% greater infection-related mortality for diabetic compared to non-diabetic kidney transplant recipients [38]. Between 2002 and 2007 when FAVORIT trial recruited 4110 renal transplant recipients, 4009 were followed to completion of the portion reviewed herein. Of the 4009, 2323 had received a non-living donor allograft while 1868 had received a living donor organ. Of the 2323 non-living allograft recipients, 871 (37.5%) were taking a calcium channel blocker while 1452(62.4%) were not taking a calcium channel blocker. Of the 1686 living donor organ recipients, 531 (31.5%) were taking a calcium channel blocker while 1155 (68.5%) were not taking a calcium channel blocker. Kaplan-Meier survival curves demonstrated no benefit for patient or allograft survivals over six years of follow-up. This is approximately nine years post-transplant surgery, a point at which both patient and allograft survival rates were not less than 75% for several sub-groups [39]. We have not analyzed this data according to the impact of statin medication upon groups with or without use of calcium channel blockers. Statins appear to inhibit a process by which excess growth of vascular smooth muscle cells pathologically associated with a subendothelial collection of matrix metalloprotein and LDL cholesterol. Studies also indicate that statins activate an L-type calcium that can be antagonized by calcium channel blockers, thus amlodipine cooperates in this statin end-point [40].

Rescue of neutrophilic phagocytic function through use of nifedipine among hemodialysis patients has been reported [41]. When calcium channel blockers were tested for protection from infections in the intra-venous tubing used as temporary or permanent access for hemodialysis, there was no clinical benefit reported [42]. Another instance of failure to find benefit from calcium channel blockers in the infection risk situation, occurred with use of verapamil for the first ten post-operative days following kidney transplantation [43]. Among 152 renal transplant recipients 16 of 77 (21%) who received 240 mg of verapamil for the first ten post-operative days developed a significant infection over the next 2–6 months compared with 4 of 75 (5%) who did not. 20 patients were hospitalized for infection in this brief follow up, 4 died (20%), including 3 of the 16 (19%) who had received verapamil vs. 1 of 4 (25%) who did not receive verapamil. However, episodes of infection occurred 6–20 weeks after verapamil, leave some biologic doubt as to causality. Despite levels of cyclosporine being higher during verapamil treatment, the occurrence of biopsy -proven allograft rejection was not significantly different between the two groups: 19/77 (25%) for the verapamil group vs. 22/75 (29%) for the no-verapamil group. The incidence of acute graft dysfunction, however, from a non-rejection cause (acute tubular necrosis) was lower in the verapamil group: 9/77 (12%) vs. 31/75 (41%) in the no-verapamil group. Since episodes of acute renal failure occurred in the immediate post-operative period, a statistical relationship with plausible biologic validity to verapamil is noted. And post-hoc evaluation of infection risk studies in populations with multiple risk factors are difficult to control for analysis. In a post-hoc evaluation of the FAVORIT study, which had identified infection as the single greatest long-term risk factor to survival of allograft recipients [37], there was no evidence for protection from serious infection for allograft recipients who had received calcium channel blockers vs. those who had not received calcium channel blockers [39].

## 3. Antibiotic-Resistant Pathogens May Utilize Channels Sensitive to Calcium Channel Blockers

Specific mechanisms for protection from sepsis by calcium channel blockers may include control of concentration of cytosolic calcium of mono- and poly-morphonuclear cells for efficient chemotaxis, migration, adhesion, phagocytosis [44,45] while limiting excessive cytokine response with massive capillary leaking found in the adult respiratory distress syndrome [46,47].

### 3.1. Treatment Resistance through Antibiotic Extrusion by the Pathogen or Host Cell

Ultimately, an effect on invading pathogens to limit natural selection of antibiotic-resistant strains would be a cost-effect efficiency goal (Table 2). Calcium channel blockers have been studied in quinolone-resistant pneumococcal pneumonia [48], rifampicin-resistant tuberculosis [49], quinine-resistant *Plasmodium falciparum* malaria [50,51], and praziquantel-resistant *Schistosoma mansoni* infestation [52,53], amphotericin-resistant *Leishmania donovani* [54], and eflornithine-resistant *Trypanosoma cruzi* [55]. Benidipine may be useful for treatment of *Toxoplasma gondii* infestation for individuals allergic to sulfonamides like sulfadiazine or sulfamethoxazole [56].

Pathogens able to efficiently extrude antibiotics quickly become resistant since they are being treated with progressively higher doses. Calcium channel blockers have been useful through closing the channel through which antibiotics are extruded. *Mycobacterium tuberculosis* has the capacity to extrude antibiotics which have entered through its tough outer membrane. The efflux mechanism [57] can be demonstrated following engulfing of the pathogen by macrophage. Verapamil inhibits the efflux pumping of antibiotics by closing the critical channel [58,59,60], which is not a typical calcium channel as shown by comparison with isomers and metabolites that are very effective at the extrusion channel, but ineffective at the calcium channel. 

Since treatment of resistant mycobacteria with new medications is costly, studies have utilized verapamil to achieve in vitro and in vivo eradication by bedaquiline at lower effective dose [61,62]. One of the dangerous side effects of bedaquiline, an ATP synthase inhibitor, is prolongation of QTc interval, increasing the risk for ventricular arrhythmias. Verapamil suppresses this side effect. The calcium channel blocker, nimodipine used in slow, continuous infusion has been found effective in normalizing the electrocardiographic changes found in experimental cerebral malaria [54]. Resistance to rifampicin and other antibiotics like ethambutol is a major impediment to recovery from pulmonary tuberculosis for individuals who are immune-suppressed by malnutrition, uncontrolled glycemia, uremia, cancer chemotherapy. In mice, the use of verapamil has been shown to reduce drug resistance by decreasing the extrusion of rifampicin. A similar mechanism for prevention of uncontrolled infestation with Plasmodium falciparum due to drug resistance has been described with effective use of a calcium channel blocker.

Two biologic defensive mechanisms are responsible for the antibiotic resistance of *Mycobacterium tuberculosis* and *M. smegmatis*. The first is the antibiotic efflux process which has now been demonstrated on the cell membrane where a protein is described that forms the structure of a channel with a preference for cations [63]. The second is the finding that the same protein channel which extrudes cations and antibiotics also produces a toxin which causes sufficient necrosis to allow the pathogen to escape from the macrophage which had recently engulfed it. This same channel also allows the pathogen to take up nutrients before it escapes [64]. Several gram negative and gram-positive bacteria have been found to have the potential for antibiotic efflux. Among the more well-known gram-negative bacteria are *Bacteroides*, *Brucella*, *Campylobacter*, *Enterobacter*, *haemophilus*, *Neisseria*, *Pseudomonas*, *Vibrio*. Among the more well-known gram-positive bacteria are *Bacillus*, *Clostridium*, *Listeria*, *Mycobacterium*, *Staphylococcus*. A rare *Mycobacterium*, *M. abscessus* is an important pathogen in cystic fibrosis, an inherited disorder with an increased risk of pneumonia associated with airway obstruction. Although this pathogen is fast-growing, it is frequently antibiotic-resistant [65]. Data on calcium channel blocker impact in this rare form of tuberculosis may be forth-coming from current studies, particularly in India. It is now known that antibiotic-resistant *M. tuberculosis* not only works by extrusion of antibiotics, but also by preventing their entry in the first place.

### 3.2. Other Mechanisms of Pathogen Resistance Involving Calcium Ion Channels

The normal concentration of calcium in the plasma is in milli-molar range (2 mMol/L) while that of the cytosol of both host cells [66] and invading pathogens [67] is in micromoles (0.1 micromol/L), a thousand-fold difference. In addition to this initial environmental shock, the pathogen may then be attacked by a sudden burst of intracellular calcium at an even higher concentration as the host cell attempts eradication (Table 2). Two protective responses have been demonstrated in pathogens. The first adaptive mechanism seeks to protect from an impending state of oxidative stress through generation of anti-oxidant proteins, such as catalase and superoxide dismutase. Catalase may be responsible for survival of antibiotic-resistant *P. aeruginosa* [68], but for *Bordetella pertussis*, dismutase is more important than catalase for survival within polymorphonuclear leukocytes [69]. The second adaptive mechanism involves influx of cytosolic calcium across the plasma membrane of the host [30] by means of energy generated by ATPase, which mechanism has been observed in *Bacillus anthracis*, *Clostridium perfringens*, and *Streptococcus pneumoniae* (Table 2). It would appear the first of these mechanisms would be of relatively short-term benefit to the pathogen. The second of these two would allow for a more interference with host cellular defenses. Thus, there are attempts to develop drugs that inhibit the calcium efflux transport function of the pathogen [57].

Calcium-related mechanisms have been identified in certain fungi which may be invasive to humans. *Aspergillus fumigatus*, an allergen and an invasive pulmonary pathogen, utilizes the calcium/calmodulin/calcineurin pathway [70] in colony growth through extension and branching of hyphae. Cyclosporine is a growth inhibitor. Calcium pathways are actively-employed by *Saccharomyces cerevisiae*, *Candida allicins* and *Cryptococcus neoformans*. Inhibition of growth of *C. neoformans* by the calcineurin inhibitor, tacrolimus, has been detected [71]. Inhibition of development of hyphae of *Candida albicans* (Table 2) has been reported for verapamil [72].

Measurement of cytosolic calcium of the Sprague-Dawley rat [73] or human [74] hepatocyte by the fluorescent indicator, Fura 4f, indicated a level of ≈1 micro-molar could be achieved by ATP stimulation. Thus, the normal concentration of 0.1 micro-molar would be elevated ten-fold during hepatitis virus cell invasion through a mobilization of calcium. But in a counter move, hepatitis virus pathogens may direct cytosolic calcium levels above 1 micro-molar into temporary stores in mitochondria. Since the leading intra-cellular store of calcium is endoplasmic reticulum, the first phase calcium release would occur from this store. High concentration of this cation might lead to host cell injury. An example of unregulated calcium entry into cytosol has been studied in retinal cells which are destroyed in rodents exposed to HIV-1 virus [75].

Therefore, in a second phase while a short-term bolus injection of calcium might eradicate an invading pathogen, to protect the host cell from prolonged excess calcium this cation must be removed from cytosol to either intra-cellular stores by sarco-endoplasmic reticulum ATPase pumping or to extra-cellular environment by means of plasma membrane ATPase pumping. As extra-cellular calcium intake proceeds to replenish endoplasmic reticulum stores, a temporary high-plateau of calcium concentration may be followed by a lower resting concentration (≈0.1 micro-molar).

### 3.3. Store Operated Calcium Entry (SOCE)

With the advent of CCB it became apparent that cytotoxic levels of calcium entry across the plasma membrane could be blocked with beneficial results. Entry of calcium into the cytosol from intracellular storage zones in the endoplasmic or sarcoplasmic reticulum (Store operated calcium entry, SOCE) may have therapeutic implications [76]. The juxtaposition of the sarcoplasmic endoplasmic reticulum allows for calcium signaling to control entry for calcium into the cytosol through the plasma membrane. Studies of defense mechanisms in liver cells infected with hepatitis B virus identify SOCE as being therapeutic for a short period of time. SOCE mechanisms may be activated during times of cytogenetic, inflammatory and hemodynamic instability.

In the instance of the hepatitis B patient or the victim of an attack by poliovirus [77], calcium transfer most likely occurred in the setting of a single pathogen in an uncompromised host. This pathogenesis is more clearly delineated than that found in a trauma victim with multi-organism sepsis and shock due to capillary leak, sometimes referred to as adult respiratory distress syndrome (ARDS) during which SOCE can be sudden and to a massive degree [46,47]. An instance of SOCE that can occur without invasion by a pathogen is that of cancer metastasis [78]. Inhibition of proliferation of human leiomyoma by means of calcium release from intracellular stores via voltage gated calcium channels has been shown to be enhanced by simvastatin [66].

### 3.4. Hyperglycemia and Calcium Ion Channels

A related series of observations involves activity of the cardiac ATPase enzyme that may demonstrate increased expression following exposure to the diabetes medication liraglutide, a member of the glucagon-like peptide class of anti-hyperglycemia agents [79]. Increased cardiomyocyte plasma membrane ATPase calcium pumping has been linked to initiation and acceleration of congestive heart failure symptoms thought to be due to elevated intra-cellular calcium concentration. On the other hand, several studies have shown improved heart function with use of the diabetes medications empagliflozin, canagliflozin, or dapagliflozin, members of the sodium/glucose transporter 2 (SGLT2) inhibition class of antihyperglycemic agents [80]. Reversal of elevated cardiomyocyte calcium concentration may explain the beneficial result in heart failure reported with these medications. This mechanism could apply as well to heart failure patients who do not have the problem of hyperglycemia at least on a short-term basis [81]. Infection outcome results with SGLT2 inhibition would certainly be of interest, particularly in the realm of antibiotic-resistant pathogens.

Restoration in the capacity for phagocytosis when hyperglycemia is reversed with anti-hyperglycemic agents is reproducible and confirmed. The mechanism of disarming of the neutrophil appears to have been an increase in calcium concentration in the cytosol. If the source of calcium were the external medium of the neutrophil, then the effect of nifedipine/amlodipine would have to be exerted at the plasma membrane. But if the source of cytosolic calcium were stores in the endoplasmic reticulum, then the mechanism would be the target of future research agents. Which-ever source of calcium were involved, it would appear the pathological increase in calcium concentration in the cytoplasm was brought under control by means of a calcium channel blocker. Dihydropyridine calcium channel blockers like amlodipine or nifedipine in hypertension or with sulfonylurea medications, like glyburide or glipizide, in hyperglycemia has been found in rescue of neutrophil phagocytic function [44,45]. While the untested hypothesis that use of statin for cholesterol control may lower cost of expensive antibiotics has some rational grounds due to calcium channel blocking activity it should be remembered that for the type 2 diabetes population it is this same mechanism which may be responsible for hyperglycemia due to inhibition of beta cell insulin secretion [82].

## 4. Loss of Regulation of Inflammatory Response to Infection: Mechanisms Associated with the Sepsis Shock Syndrome

An experimental model for testing the impact of calcium channel blockers on sepsis involves ligation of the cecum with puncture of the wall of the intestine. This model has demonstrated relatively longer survival if diltiazem were injected prior to the onset of septic shock [4] in association with decreased formation of oxygen radicals [5]. Specific mechanisms for protection from sepsis by calcium channel blockers include: a decrease in cytosolic calcium of inflammation mediating cells, thereby limiting excessive cytokine responses, such as occurs in the adult respiratory distress syndrome [46]; an improved capacity to combat pathogens (chemotaxis, movement, adhesion, phagocytosis) through an increase in cytosolic calcium of polymorphonuclear cells and macrophages by release from intracellular stores (endoplasmic reticulum, sarcoplasmic reticulum, mitochondria) through alternate channels while the slow L channels from the exterior are blocked [47]; and an effect on invading pathogens to limit their capacity to select strains capable of rapid development of resistance to antibiotics. More recent reports of protection from sepsis have emerged from studies of antibiotic-resistant *Myocobacteria tuberculosis*. Along these lines, one analog of a calcium channel blocker (verapamil) may be more effective than another in assisting rifampicin in its battle with *M. tuberculosis* for reasons not easily understood in terms of L-type calcium channels, but rather by efficiency of docking at a site critical to the rifampin efflux mechanism. It is entirely possible that no available calcium blocker can reverse capillary leak as a complication of infection. Newer analogs however may become available [83]. It is now known that antibiotic-resistant *M. tuberculosis* bacilli inhibit entry and accelerate extrusion of antibiotics. Control of cytosolic calcium entry or transport from internal sources has been found critical for macrophage and neutrophil defense functions. Channels from endo- and sarcoplasmic reticula may interact with trauma/infection stresses for release of calcium from relevant storage areas. The endoplasmic reticulum utilizes inositol-3-phosphate receptors while the sarcoplasmic reticulum utilizes ryanodine receptors.

### Sepsis Leads to Capillary Leaking

Understanding of the of the sepsis syndrome has shifted focus from that of an overwhelming inflammation cascade to an emphasis upon leaking of capillary fluid, resulting in hemoconcentration with diminished blood flow to vital organs and eventually into a state of shock due to hypovolemic hypotension (Table 2). This capillary leak results from endothelial dysfunction secondary to changes in the angiopoietin system. Ordinarily, angiopoietin 1 maintains the vascular barrier, but the effect of bacterial invasion is to enhance the concentration of angiopoietin 2 through its release from storage sites in endothelial cells mediated by tumor necrosis factor alpha (TNF α). A higher concentration of angiopoietin 1 vs. angiopoietin 2 normally operates the vascular barrier through a growth factor receptor, Tie2, which is inhibited when angiopoietin 2 concentration increases. So, a marker for the severity of the state of sepsis would be the rising level of circulating angiopoietin 2, a for-warning of imminent hypovolemia due to interstitial space accumulation of electrolyte/protein-containing fluid with strong osmolar capacity. Although the local presence of angiopoietin 2 at the portal of entry may be beneficial as a way of washing out the invasive pathogen, the target of sepsis-related research would be counter-measures by which a prolonged elevated level of circulating angiopoietin 2 might be brought into balance [84,85]. Calcium entry into endothelial cells under stress appears to occur via T-type calcium channels rather than L-type. Thus, amlodipine might have no effect upon capillary leak while flunarizine might be highly effective. In addition, flunarizine has been shown to inhibit vasoconstriction by norepinephrine and to diminish the risk of small vessel thrombosis [86]. Angiotensin caused vasoconstriction can damage muscular layers of larger and endothelium of smaller blood vessels [18]. The result may be a loss of resistance to sepsis [19,20].

## 5. Vitamin D, Parathyroid Hormone, Fibroblast Growth Factor, and Klotho Interact with Sepsis Defense Mechanisms in Which Movement of Calcium and Phosphorus Are Part of the Process

Given various findings of calcium movement inside host defense cells (polymorphonuclears, monocytes, macrophages), the issue of interaction between Vitamin D treatment for protection is an obvious area of sepsis study since lack of vitamin D is associated with risk of pneumonia [87,88]. In this connection relevant roles of vitamin D consist of assisting parathyroid hormone in the absorption of calcium in the intestinal tract as well as an independent capacity in the absorption of the companion anion phosphate which does not appear to be a target for parathyroid hormone. This increased prevalence of upper respiratory tract infections has been noted in patients with low vitamin D levels without [87,88] or with [89,90] advanced kidney failure. Intensive Care Unit patients who are septic have been found to have significantly lower concentrations of Vitamin D binding protein than those who were not septic. And there was also a positive correlation between Vitamin D and the toll-like receptors needed for expression of cathelicidin LL-37 levels [25]. Cathelicidins and defensins are antimicrobial peptides which cooperate in protection from infection. Since these agents are found in bronchopulmonary sites under attack from pathogens, such as *Mycobacterium tuberculosis*, they can be expected to offer a measure of protection in the lung.

Genes are being identified which code for peptides from pathogens, setting in motion expression of receptors (called Toll-like) which assist in generation of cathelicidins [25]. Susceptibility to tuberculosis is associated with vitamin D deficiency as well as a lack of production of cathelicidins. Efficiently activated monocytes will be capable of producing cathelicidin protein plus advancing 25(OH) vitamin D to the intracellular active form 1,25(OH)_2_ D [25]. Downstream from the Vitamin D receptor is the peptide, cathelicidin, with bactericidal capacity against *Mycobacterium tuberculosis* (Table 2). This suggests that hydroxylation of the cholecalciferol by the 1-alpha hydroxylase activates cathelicidin through toll like receptor activity. Raising the question as to whether correction of Vitamin D deficiency directly confers bactericidal potential. A question to be answered is the relationship between intracellular levels of calcium, vitamin D, and calcium channel blockers. The relationship of calcium channel blockers to expression of bactericidal proteins, cathelicidin and Beta-defensin in association with intracellular elaboration of 1,25(OH)_2_ vitamin D3 remains to be fully elucidated.

Since the demonstration of lower levels of 25(OH) vitamin D, vitamin D binding protein, and cathelicidin (LL37) in the serum of critically patients in the ICU compared to healthy controls, details of these observations have become of interest in trauma centers (Table 2). Eleven healthy controls were compared to 25 non-septic ICU control subjects versus 24 septic ICU study subjects. The individuals with sepsis had significantly higher levels of BUN and creatinine with lower levels of serum albumin, consistent with acute kidney injury. There was a positive correlation between 25(OH) vitamin D and cathelicidin (LL-37) levels [89]. Filtered 25(OH) vitamin D in complex with vitamin D binding protein can be resorbed from the glomerular filtrate with the assistance of proximal tubular protein, megalin [91] while the binding protein is degraded, the 25(OH) D is converted into 1,25(OH)2 D, and both 25(OH) D + 1,25(OH)_2_ D are resorbed into the circulation. Retention of phosphorus with lowering of calcium (despite reflex increases in parathyroid hormone) has been shown to be related to mortality [92]. Kidney failure with vitamin D deficiency can generate fibroblast growth factor to assist in excretion of retained phosphorus if vitamin D is replaced [93]. Klotho, the anti-aging gene seems to have a role in lowering the elevated level of phosphorus in kidney failure in cooperation with Fibroblast Growth Factor 23. Some insight into renal failure as a risk factor for infection might be gained (Table 2) by studying response to phosphorus retention in the setting of combined effects of parathyroid hormone, fibroblast growth factor 23, and Klotho [94] cooperating to exclude excess phosphorus at the sodium/phosphate co-transporter in the proximal tubule. But while PTH and FGF 23 are capable of cooperating at the proximal tubular site for phosphate transport to decrease phosphorus resorption, they are simultaneously capable of competing with each other at a different site where PTH assists in activation of 1-alpha hydroxylase (Table 2) for completion of synthesis if 1,25(OH)_2_ cholecalciferol (calcitriol), while inhibition of 1-alpha hydroxylase by Fibroblast Growth Factor is occurring simultaneously by way of inhibiting excess intestinal absorption of calcium in the setting of risk for deposition of calcium phosphorus in vascular tissues (calciphylaxis). In terms of infection risk, if the function of white blood cells is inhibited at low levels of both phosphorus and vitamin D, then the suggestion might be that kidney dysfunction and infection are more closely associated with Fibroblast Growth Factor 23 than with parathyroid hormone.

## 6. Conclusions

It is doubtful that sponsors can be found to support placebo-controlled trials for long-term calcium channel blockade in immunosuppressed individuals (such as solid organ transplant recipients) for the purpose of determining whether there is a beneficial effect on non-cardiovascular health care outcomes. However, there are transplant registries and multicenter trial databases from which additional information might be uncovered. An effort should be made to elucidate whether benefits or risks associated with calcium channel blockers in transplant populations as an initial example of immunosuppressed study groups with equally well-recorded information. Since long-term risk of infection in recipients of kidney transplant allografts is more important than cardiovascular complications [37] and outcome results for protection from sepsis by calcium channel blockers are in conflict [3,39] further studies need to be explored in terms of the multiple mechanisms reviewed herein. With respect to other populations immunosuppressed either iatrogenically or by virtue of underlying disease, we consider that further observations suggesting that this inexpensive class of medications might enhance treatment of infectious diseases should direct future trials. Another untested possibility is the hypothesis that use of statins to lower doses and cost of expensive antibiotics [95] may be effective since these drugs also exhibit some calcium channel blocking activity [96].

## Figures and Tables

**Table 1:** Pathophysiologic Interactions of calcium ion channels with the production of pathogen mediated sepsis syndrome: Potential for therapeutic interference with calcium ion channels.

**Pathogenic Organism**	**Calcium Ion Channel Effect**
Organ specific toxicity	Envelope protein increases toxic level of cytosolic calcium in host cell
Cell membrane or wall	Antibiotic efflux may be diminished by calcium channel blockade
Organism replication	Interference with calcium dependent RNA transcription
Cytoplasm (mitochondria)	Potential to interrupt intracellular calcium shifts and interrupt calcium efflux
**Host Defense**	**Calcium Ion Channel Effect**
Immunocompetence	Calcium activated potassium channels regulate Lymphocyte activation Mitogenesis Cell volume
Cellular Immunity Permeability, necrosis, apoptosis	Release of intracellular calcium leads to mitochondrial permeability and influx of extracellular calcium and permeability, necrosis, apoptosis
Humoral Immunity	Calcium controls antibody formation
Inflammasome	Calcium has a role in the production of TNF alpha, IL-1 beta

**Table 2:** Calcium Ion Channels and hypothetical role in expression of infections.

I. Relationship of clinical infections to calcium channels (retrospective studies) Pneumonia: acute infection without antibiotic resistance 1. General population 2. Recipients of Kidney Transplant allografts a. Positive outcome results with calcium channel blockers b. Negative outcome results with calcium channel blockers Sepsis with or without antibiotic-resistant pathogens 1. Potential benefit of calcium channel blockers in combination with more expensive drugs in an attempt to reduce costs while minimizing side effects of higher doses a. quinolone-resistant streptococcus b. rifampicin-resistant mycobacterium c. quinine-resistant plasmodium d. praziquantel-resistant schistosome e. amphotericin-resistant leishmania f. eflornithine-resistant trypanosome
II. Role of calcium movement in white blood cell defense against pathogens Neutrophils and macrophages may benefit from calcium channel blockers Restoration of capacity to attack pathogen Limitation of capacity of pathogen to extrude antibiotic
III. Sepsis with shock following trauma Intracellular calcium movement from storage sites may be stabilized by calcium channel -blockers, limiting cell injury B. Capillary leak during sepsis1. Angiopoietin 2 as a factor in sepsis-related capillary leaking Flunarizine, which blocks both calcium influx and calcium movement from intracellular stores, in prevention of angiopoietin 2—related capillary leaking
IV. Pathogen colony growth mechanisms may or may not involve calcium Generation of anti-oxidants (catalase, dismutase) Bortadella pertussis Pseudomonas aeruginosa Efflux of calcium from host cells Bacillus anthracis Clostridium perfringens Streptococcus pneumoniae Colony growth mechanisms inhibited by calcium channel blockers Aspergillus fumigatus Saccharomyces cerevisiae Candida albicans Cryptococcus neoformans
V. Mechanisms of Calcium balance in kidney failure Vitamin D (and cathelicidin) deficiency association with infection risk Promotes availability of calcium through intestinal absorption Promotes bone calcification at the growing front of osteoid Parathyroid hormone excess promotes lysis of calcified bone with excess calcium/phosphorus that may deposit in soft tissue, including blood vessels with skin cellulitis (calciphylaxis). Inhibits resorption of phosphate in kidney proximal tubule. Promotes activity of 1-alpha hydroxylase in renal proximal tubule, which converts 25(OH) Vitamin D to its most active form 1,25(OH)_2_ vitamin D. Fibroblast Growth Factor 23 minimizes the accumulation of phosphate which is associated with an increased mortality rate. Cooperates with parathyroid hormone in the inhibition of resorption of phosphate in kidney proximal tubule. Competes with parathyroid hormone’s action on 1-alpha hydroxylase in the proximal tubule to generate the most active form of Vitamin D. Klotho, the antiaging gene, cooperates with parathyroid hormone and fibroblast growth factor 23 by inhibiting phosphate resorption in the kidney proximal tubule.
